# A novel de novo *PAX6 *mutation in an Ashkenazi-Jewish family with aniridia

**Published:** 2008-01-28

**Authors:** Dikla Bandah, Ada Rosenmann, Anat Blumenfeld, Edward Averbukh, Eyal Banin, Dror Sharon

**Affiliations:** Department of Ophthalmology, Hadassah-Hebrew University Medical Center, Jerusalem, Israel

## Abstract

**Purpose:**

To report a novel de novo *PAX6* mutation in an Ashkenazi-Jewish family with autosomal dominant aniridia.

**Methods:**

A mother and her daughter of Ashkenazi-Jewish origin were diagnosed with aniridia. Blood samples were drawn from family members and DNA was analyzed by direct sequencing and microsatellite marker analysis.

**Results:**

The index patient and her daughter were affected with aniridia accompanied by congenital cataract, nystagmus, and glaucoma. A heterozygous *PAX6* frameshift mutation in exon 6 (c.577_578insG, insG@Gly72) was identified in the affected individuals and not in any of the unaffected family members including the parents of the index patient. Microsatellite analysis revealed that the index patient inherited the disease haplotype from her unaffected father. A sequence analysis of human *PAX6* expressed sequence tags revealed the identification of spliced transcripts initiating from introns 4, 6, 7, 8, and 11.

**Conclusions:**

A novel de novo frameshift mutation in *PAX6*, which presumably occurred in the paternal gamete, was found in a family with autosomal dominant aniridia. The location of the mutation suggests that only full-length PAX6 isoforms would be disrupted, indicating that the normal expression of shorter, paired-less, protein isoforms cannot prevent manifestation of the disease.

## Introduction

Aniridia (OMIM 106210) is an inherited developmental panocular disorder characterized by iris hypoplasia and is usually accompanied by glaucoma, cataract, corneal opacity, and foveal hypoplasia. The disease is mostly inherited in an autosomal dominant mode with variable expressivity, and about one-third of the patients are sporadic.

Autosomal dominant aniridia is caused by various heterozygous mutations in the paired box gene-6 (*PAX6*) [1,2], which encodes a transcription factor playing a major role in developmental processes in several organs including the eye [3,4]. Classic experiments have shown that knocking-out *PAX6* from the drosophila or mouse genome results in the absence of the eye [5,6]. Heterozygous *PAX6* mutations in humans can also lead to a variety of ocular abnormalities such as congenital cataract [7] and Peter's anomaly [8]. A recent genotype-phenotype correlation analysis of 286 pathogenic *PAX6* mutations revealed that mutations that introduce a premature termination codon into the open reading frame are predominantly associated with aniridia [9].

A relatively large number of *PAX6* mRNA sequences and protein isoforms produced by alternative transcripts have been reported in different species [10-15]. Some of these are named paired-less since the encoded protein isoform does not contain the paired domain which is very important for the function of the PAX6 protein.

We present here a de novo and novel *PAX6* frameshift mutation that causes aniridia in an Ashkenazi-Jewish family. In addition, we performed sequence analysis of the human *PAX6* expressed sequence tags (ESTs) and identified transcripts that are likely to encode paired-less protein isoforms. We suggest that the normal expression of the paired-less isoforms does not prevent the expression of the aniridia phenotype.

## Methods

Two members of an Ashkenazi-Jewish family with aniridia were identified. Over the years, repeated ophthalmologic examinations were performed including best corrected visual acuity, refraction, intraocular pressure, slit lamp examination, and funduscopy. Both affected patients also underwent surgical procedures as detailed below. The tenets of the Declaration of Helsinki were followed, and informed consent was obtained from all patients who participated in this study before any donation of a blood sample. Blood samples were obtained from the index patient, her affected daughter, and unaffected family members for DNA analysis. Genomic DNA was extracted from peripheral blood of all family members using the FlexiGene DNA kit (Qiagen, Hilden, Germany). Microsatellite analysis using two markers (D11S904 and D11S935) flanking *PAX6 *was performed using the ABI PRISM 3700 system (Applied Biosystems, Foster City, CA). Mutation analysis of *PAX6* was performed by direct sequencing of polymerase chain reaction (PCR) products.

The *PAX6* mutation identified in this study has been deposited in the Human PAX6 Allelic Variant database (accession number 0000000367) [16]. To identify *PAX6* GenBank entries that contain intronic sequences, we performed a BLAST analysis of each *PAX6* intron against all available human ESTs. Splice-site sequence analysis was performed using the Splice Site Prediction by Neural Network with a cutoff parameter of 0.1.

## Results

### Clinical description

**Figure 1:**
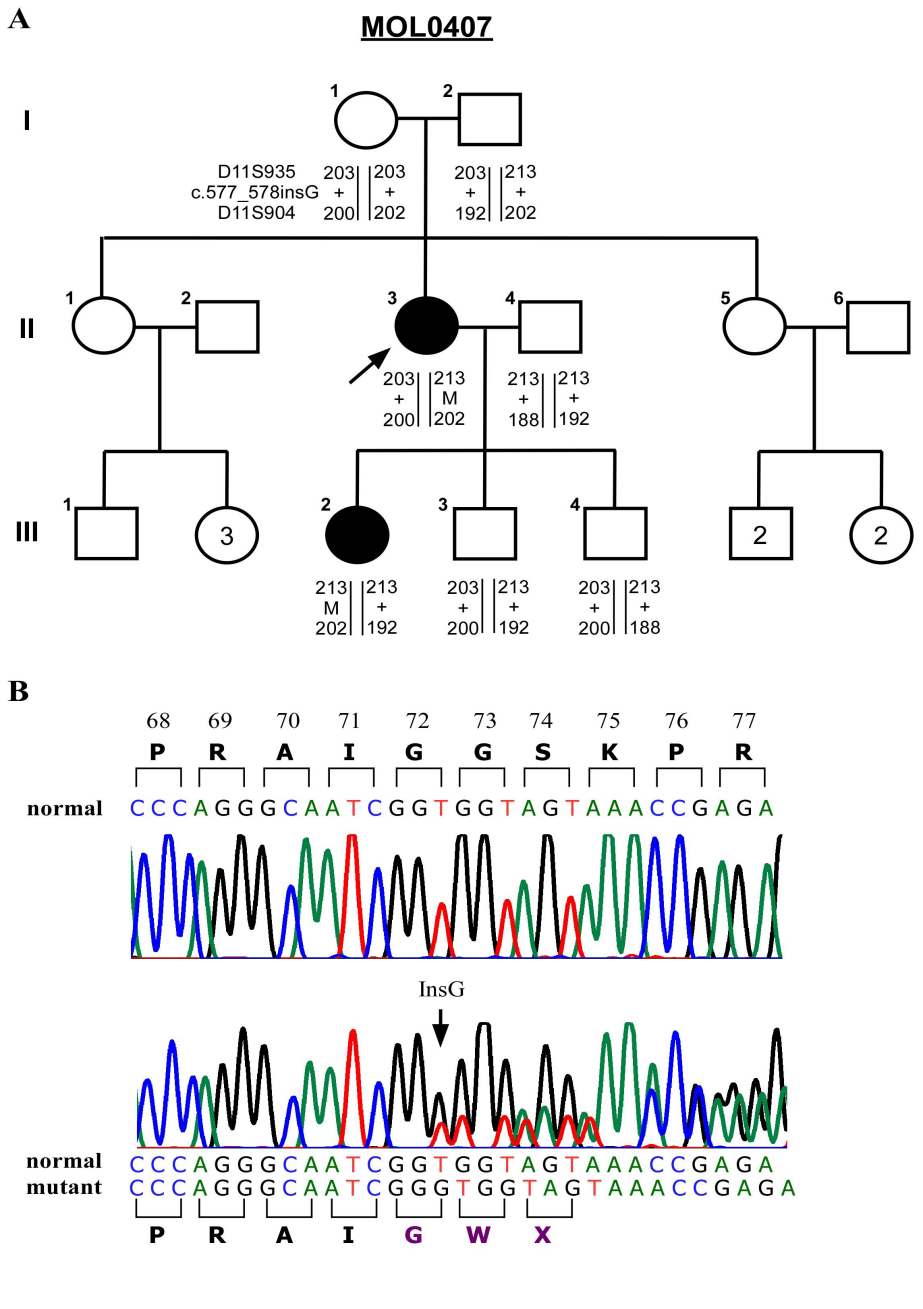
A novel de-novo *PAX6* mutation in an Ashkenazi-Jewish family. **A:** A schematic representation of family MOL0407 is shown. The black filled shapes denote individuals affected with aniridia. The numbers above the symbols indicate the recruited individuals, and the numbers within the symbols indicate the number of siblings. The arrow indicates the index patient. The haplotype of each individual is represented below the individual symbol. Numbers represent the size of the PCR product containing the microsatellite sequence, "M" represents the mutant allele, and "+" represents the normal allele. **B:** Sequence analysis of exon 6 in *PAX6* is shown. The upper chromatogram represents the wild-type sequence of an unaffected family member, and the lower chromatogram represents a heterozygous mutation (patient II-3). The position of the insertion is indicated by an arrow. The wild type PAX6 amino acid sequence is shown above the upper chromatogram and the mutant amino acid sequence is shown below the lower chromatogram.

A family from an Ashkenazi-Jewish ancestry (Figure 1A) was recruited for this study. The index case (II-3) had no family history of visual impairment and manifested total absence of the iris in the right eye, along with a large iris coloboma in the left eye. She was also diagnosed with congenital cataract, nystagmus, glaucoma, corneal dystrophy, and spherophakia. Her refractive error was −4.5/-2.0x10° (right) and −2.0/-1.25x180° (left). Her visual acuity (VA) was 6/60 at age 9 and 11 and dropped to counting fingers (FC) at 30 cm at age 49. On examination at age 49, intraocular pressure was 27 mmHg in the right eye (RE) and 25 mmHg in the left eye (LE). She was not receiving treatment at this time. Corneas were vascularized in both eyes (BE). Anterior chambers were deep, total aniridia was evident in the RE, large iris coloboma was evident in LE, and remnants of lens in BE (patient did not undergo previous cataract surgery). Fundus details were difficult to see, but retinas were flat per ultrasound. Her daughter (III-2) was diagnosed at the age of two months with aniridia, congenital cataract, nystagmus, glaucoma, and lack of a macular reflex. She underwent a filtration procedure at the age of three months and was treated with various topical anti-glaucoma medications over the years. Refraction at five years of age was −1.5/-0.75x180° (right) and −1.5/-0.50x180° (left). Her VA was 5/60 (RE) and 5/36 (LE) at the age of 14 years. At the age of 24 years she underwent cataract surgery in both eyes, and at the age of 26 the intraocular lens (IOL) in the RE was repositioned. Her VA dropped to FC 1 m (RE) and FC 2 m (LE) by the age of 30. At that time, intraocular pressure was 31 mmHg in the RE and 21 mmHg LE. There was nystagmus and in both eyes the corneas were vascularized, with poor epithelialization. Aniridia was noted in both eyes with some peripheral remnants of iris adherent to the lens capsules. IOLs were positioned in the sulcus in both eyes. Fundus details were difficult to see, but significant cupping of both optic discs (0.7–0.8) was noted. Treatment with Travoprost was initiated in an attempt to lower intra-ocular pressure.

### Molecular analysis

Mutation analysis of *PAX6* in the index patient revealed a heterozygous novel frameshift insertion (c.577_578insG) within the paired domain in exon 6 (Figure 1B). Her affected daughter was also found to be heterozygous for this mutation. However, none of her unaffected parents carried this mutation. Aiming to study the inheritance of the haplotype carrying the mutation, we genotyped two microsatellite markers (D11S904 and D11S935) flanking *PAX6* in available family members (Figure 1A). Both affected individuals (II-3 and III-2) shared the disease-related haplotype (213–202) while none of the unaffected siblings of III-2 inherited this haplotype. The index patient inherited the disease-related haplotype from her unaffected father who did not carry the causative mutation. Aiming to verify paternity, we genotyped four additional microsatellite markers located on different autosomes (data not shown) and verified that individual I-2 is indeed the biological father of II-3.

### Sequence analysis of *PAX6*-related expressed sequence tags

**Figure 2:**
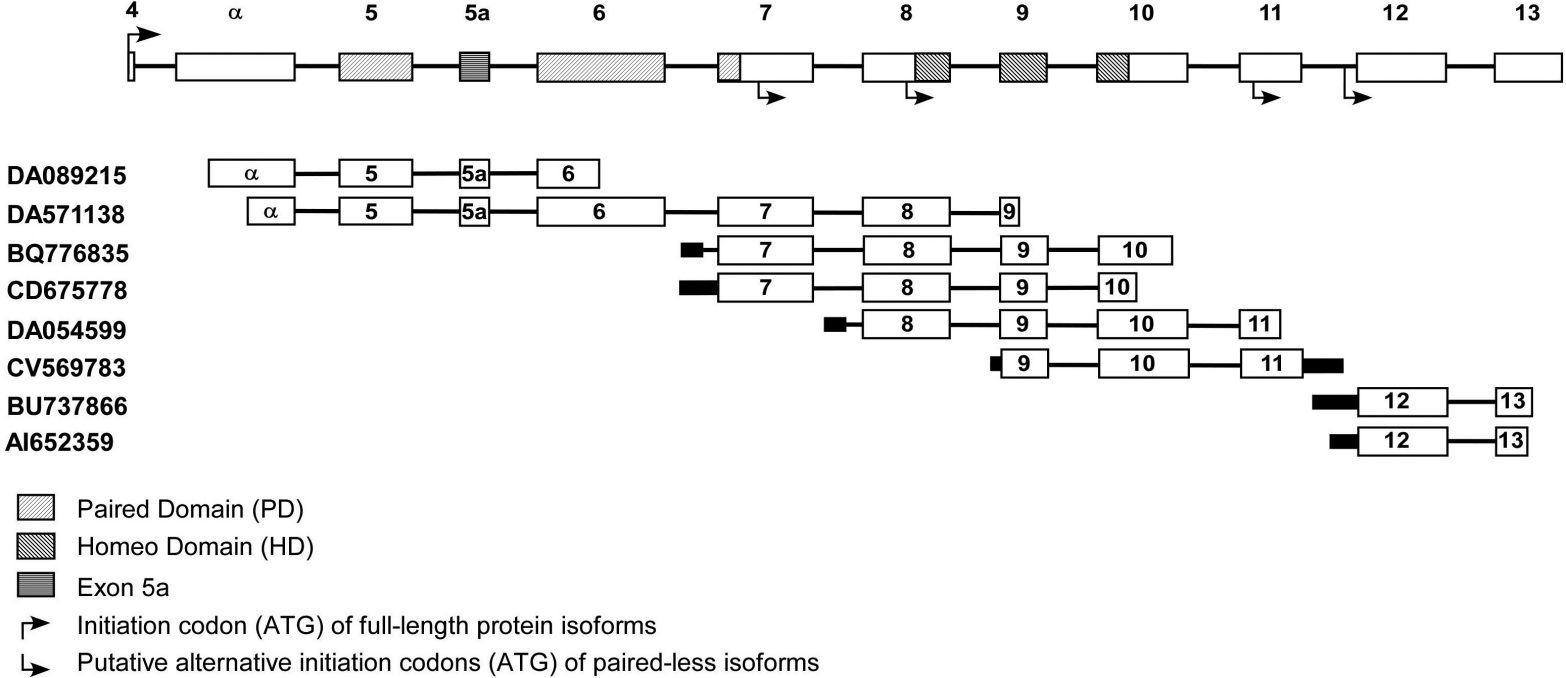
A schematic representation of the human *PAX6* and expressed sequence tags (ESTs) initiating from intronic regions. The upper panel illustrates the structure of the human *PAX6*. Only the genomic region containing the coding exons is depicted with arrows indicating the location of the initiation codons. The regions covered by each of the eight spliced ESTs containing intronic sequences are depicted below. Spliced out regions are illustrated as a thin line.

To determine whether paired-less transcripts are produced by the human *PAX6*, we performed a detailed analysis of *PAX6* ESTs deposited in GenBank. We used BLAST analysis to identify *PAX6* ESTs that contain intronic sequences and studied the composition of each of these sequences. We identified 28 non-characterized ESTs with full or partial sequences of introns 4, 6, 7, 8, 9, 10, 11, and 12. A detailed analysis of these sequences revealed 20 ESTs with no evidence for splicing events (data not shown). These sequences were excluded from subsequent analyses since they are likely to represent genomic contamination and not true mRNA sequences. The remaining eight ESTs can be divided by the intron from which the putative alternative transcription starts: two ESTs from the evolutionary-conserved alpha promoter/enhancer within intron 4 [17], two from intron 6, one from intron 7, one from intron 8, and two from intron 11 (Figure 2). An analysis of splicing events in these sequences revealed four sites in which alternative splicing occurred in the human *PAX6*, some of which have been previously described in mammalian and non-mammalian species. The two ESTs initiating from the alpha promoter/enhancer share the same splicing event (a splice site score of 0.17). One of the intron 6 ESTs (BQ776835) contains a strong novel splice site (score of 0.85) while the second EST (CD675778) shows no splicing event within intron 6. A fourth novel splicing event was identified within intron 7 (score of 0.30). The remaining ESTs do not show intronic splicing events but do contain splicing at the canonical *PAX6* splice sites and are therefore regarded as true mRNA transcripts. All eight EST sequences initiating from intronic regions are expected to encode short paired-less PAX6 isoforms with an open reading frame starting within exon 7, exon 8, exon 11, or intron 11 (Figure 2)

## Discussion

The identified novel mutation in *PAX6*, which occurred de novo in our index patient, is likely to result in a transcript that is recognized by the nonsense-mediated mRNA decay (NMD) system [18] and therefore will be degraded, resulting in a 50% reduction of the full-length PAX6 protein. This result is in-line with the suggested genotype-phenotype correlation analysis of *PAX6* [9], showing that frameshift and nonsense mutations usually result in the aniridia phenotype.

A large proportion of genes in the human genome encode more than one protein isoform through alternative splicing or alternative initiation of transcription. *PAX6* might be one of the extreme cases in which multiple alternative promoters and multiple alternative splice-sites generate a large number of protein isoforms [14,15,19]. One of the most intriguing questions regarding *PAX6* is: What is the function, if there is one, of the short, paired-less, isoforms? The existence of short mRNA transcripts transcribed from *PAX6* has been described in a variety of species including those initiating from introns 4, 6, 7, and 11 [14,15,20]. Some of these transcripts have been shown to be translated to paired-less PAX6 isoforms and in some cases, have a unique expression pattern, which is highly conserved along evolution [19]. Overexpression of a paired-less isoform in the mouse retina revealed a microphthalmic phenotype [15]. These observations suggest that the paired-less isoforms have a function that is different from the current function attributed to the PAX6 protein. However, the existence of such transcripts was not studied in humans. Our analysis of all *PAX6* ESTs as presented here shows that alternative promoters are likely to be active in the human *PAX6* within introns 4, 6, 7, and 11 as previously described in other species [14,15,19]. In addition, one EST sequence indicates that another alternative promoter might be located within intron 8. The predicted amino acid sequence of these transcripts revealed that all ESTs initiated within introns 4 and 6 result in the same protein isoform while shorter isoforms might be produced by the transcripts initiating from introns 8 and 11. Nonetheless, all of these isoforms do not contain the paired region in their open reading frame.

The c.577_578insG mutation identified here (as well as other null mutations previously reported in exon 6) are not expected to disrupt these shorter isoforms since exon 6 is not part of their open reading frame [14,15,20]. Thus, these isoforms are probably expressed from the mutant allele even without the expression of the full-length protein. Our results indicate that the mechanism by which null *PAX6* mutations cause aniridia might be either haploinsufficiency of the full-length PAX6 protein or an aberrant ratio of full-length to paired-less protein isoforms.

## References

[1] Glaser T, Walton DS, Maas RL (1992). Genomic structure, evolutionary conservation and aniridia mutations in the human PAX6 gene.. Nat Genet.

[2] Jordan T, Hanson I, Zaletayev D, Hodgson S, Prosser J, Seawright A, Hastie N, van Heyningen V (1992). The human PAX6 gene is mutated in two patients with aniridia.. Nat Genet.

[3] van Heyningen V, Williamson KA (2002). PAX6 in sensory development.. Hum Mol Genet.

[4] Simpson TI, Price DJ (2002). Pax6; a pleiotropic player in development.. Bioessays.

[5] Hill RE, Favor J, Hogan BL, Ton CC, Saunders GF, Hanson IM, Prosser J, Jordan T, Hastie ND, van Heyningen V (1991). Mouse small eye results from mutations in a paired-like homeobox-containing gene.. Nature.

[6] Quiring R, Walldorf U, Kloter U, Gehring WJ (1994). Homology of the eyeless gene of Drosophila to the Small eye gene in mice and Aniridia in humans.. Science.

[7] Hanson I, Churchill A, Love J, Axton R, Moore T, Clarke M, Meire F, van Heyningen V (1999). Missense mutations in the most ancient residues of the PAX6 paired domain underlie a spectrum of human congenital eye malformations.. Hum Mol Genet.

[8] Hanson IM, Fletcher JM, Jordan T, Brown A, Taylor D, Adams RJ, Punnett HH, van Heyningen V (1994). Mutations at the PAX6 locus are found in heterogeneous anterior segment malformations including Peters' anomaly.. Nat Genet.

[9] Tzoulaki I, White IM, Hanson IM (2005). PAX6 mutations: genotype-phenotype correlations.. BMC Genet.

[10] Walther C, Gruss P (1991). Pax-6, a murine paired box gene, is expressed in the developing CNS.. Development.

[11] Dozier C, Carriere C, Grevin D, Martin P, Quatannens B, Stehelin D, Saule S (1993). Structure and DNA-binding properties of Pax-QNR, a paired box- and homeobox-containing gene.. Cell Growth Differ.

[12] Jaworski C, Sperbeck S, Graham C, Wistow G (1997). Alternative splicing of Pax6 in bovine eye and evolutionary conservation of intron sequences.. Biochem Biophys Res Commun.

[13] Carriere C, Plaza S, Martin P, Quatannens B, Bailly M, Stehelin D, Saule S (1993). Characterization of quail Pax-6 (Pax-QNR) proteins expressed in the neuroretina.. Mol Cell Biol.

[14] Bandah D, Swissa T, Ben-Shlomo G, Banin E, Ofri R, Sharon D (2007). A Complex Expression Pattern of Pax6 in the Pigeon Retina.. Invest Ophthalmol Vis Sci.

[15] Kim J, Lauderdale JD (2006). Analysis of Pax6 expression using a BAC transgene reveals the presence of a paired-less isoform of Pax6 in the eye and olfactory bulb.. Dev Biol.

[16] Brown A, McKie M, van Heyningen V, Prosser J (1998). The Human PAX6 Mutation Database.. Nucleic Acids Res.

[17] Xu ZP, Saunders GF (1998). PAX6 intronic sequence targets expression to the spinal cord.. Dev Genet.

[18] Hentze MW, Kulozik AE (1999). A perfect message: RNA surveillance and nonsense-mediated decay.. Cell.

[19] Lakowski J, Majumder A, Lauderdale JD (2007). Mechanisms controlling Pax6 isoform expression in the retina have been conserved between teleosts and mammals.. Dev Biol.

[20] Kleinjan DA, Seawright A, Childs AJ, van Heyningen V (2004). Conserved elements in Pax6 intron 7 involved in (auto)regulation and alternative transcription.. Dev Biol.

